# Postoperative Blood Glucose Spikes Predict Morbidity After Pancreatoduodenectomy

**DOI:** 10.1155/jdr/3576207

**Published:** 2026-06-22

**Authors:** Shigeyuki Nagata, Seigo Korematsu, Takashi Maeda, Hiroyuki Orita, Daisuke Korenaga

**Affiliations:** ^1^ Department of Surgery, Iizuka Hospital, Fukuoka, Japan; ^2^ Department of Surgery, Nakatsu Municipal Hospital, Oita, Japan; ^3^ Department of Surgery, Hiroshima Red Cross Hospital & Atomic Bomb Survivor Hospital, Hiroshima, Japan, jrc.or.jp; ^4^ Department of Pediatrics, Saitama Medical Center, Saitama, Japan, saitama-med.ac.jp

**Keywords:** blood glucose, continuous glucose monitoring, morbidity, pancreatectomy, postoperative complications

## Abstract

**Background:**

Pancreatoduodenectomy and distal pancreatectomy are standard surgical treatments for pancreatic tumors but are associated with significant postoperative morbidity. A blood glucose spike (BGS), defined as a rapid, transient rise in glucose levels, may impair vascular function and affect recovery. We aimed to characterize postoperative BGS using continuous glucose monitoring and evaluate associated complications.

**Methods:**

This prospective observational study included 46 patients who underwent pancreatoduodenectomy (*n* = 34) or distal pancreatectomy (*n* = 12) between December 2017 and January 2023 at two centers. Glucose levels were continuously monitored for up to 14 days postoperatively using continuous glucose monitoring. BGS was defined as a glucose rise of > 7.8 mmol/L (140 mg/dL), followed by a drop to < 7.8 mmol/L within 2 h. Complications were graded using the Clavien–Dindo classification, with Grade ≥ II considered clinically significant. Multivariate logistic regression identified predictors of morbidity.

**Results:**

Patients who underwent pancreatoduodenectomy had lower preoperative albumin levels, prognostic nutritional index values, and more frequent nocturnal BGS (*p* = 0.009) than did those who underwent distal pancreatectomy. Mean, maximum, and minimum glucose levels did not differ between groups. Postprandial BGS occurred in all patients after distal pancreatectomy. BGS was associated with complications (*p* < 0.001) and was the strongest predictor of postoperative morbidity (*p* = 0.023) in those who underwent pancreatoduodenectomy.

**Conclusion:**

Postoperative BGS is common after pancreatectomy and strongly predicts morbidity after pancreatoduodenectomy. Continuous glucose monitoring–based detection and management of BGS may reduce postoperative complications.

## 1. Introduction

Pancreatoduodenectomy (PD) and distal pancreatectomy (DP) are well‐established procedures for pancreatic and periampullary tumors. Despite advances in surgical techniques and perioperative care, PD remains associated with a high risk of severe complications, including pancreatic fistula, hemorrhage, and infection [1–3].

Blood glucose levels fluctuate naturally throughout the day, particularly around meals. While most variations are gradual, some present as rapid and transient rises, referred to as blood glucose spikes (BGSs). These spikes may result from impaired insulin secretion, which is often associated with aging, obesity, or pancreatic insufficiency. BGS has been associated with endothelial dysfunction, atherosclerosis, cardiovascular events [4–6], and increased cancer‐related mortality [7], indicating that it may adversely affect postoperative recovery.

Continuous glucose monitoring (CGM) enables detailed evaluation of glycemic variability and is increasingly employed in patients with unstable glycemic profiles. Previous studies have shown that BGS occurs more frequently after gastrectomy with Roux‐en‐Y or Billroth I reconstruction than after pylorus‐preserving procedures [8]. However, the frequency and clinical significance of these complications following pancreatic surgery remain unclear.

The aims of this study were to characterize postoperative glycemic fluctuations using CGM in patients who underwent PD or DP and to determine the association between BGS and postoperative complications. We hypothesized that postoperative BGS is independently associated with postoperative complications after pancreatectomy.

## 2. Methods

### 2.1. Study Design and Patient Selection

This prospective observational study included patients who underwent either PD or DP between December 2017 and January 2023 at Nakatsu Municipal Hospital and the Hiroshima Red Cross Hospital & Atomic Bomb Survivors Hospital. Patients were eligible for inclusion if they met all of the following criteria: (1) were scheduled to undergo PD (open approach) or DP (either open or laparoscopic approach) for diseases of the biliary tract, including the duodenal papilla, or for benign or malignant pancreatic diseases; (2) had an Eastern Cooperative Oncology Group Performance Status of 0 or 1; (3) were aged 20 years or older at the time of providing informed consent; and (4) demonstrated adequate function of major organs, including the heart, lungs, liver, and kidneys, indicating that they were medically fit for surgery. Exclusion criteria were total pancreatectomy, combined major organ resections (with the exception of cholecystectomy or splenectomy), and inability to provide informed consent.

### 2.2. Surgical Procedures

All PD procedures were performed via open surgery, with standard lymph node dissection and reconstruction using the modified Child method [9]. Pancreatojejunostomy was conducted using the modified Blumgart technique [2]. A lost stent was placed in the main pancreatic duct in all cases. DP was performed with a splenectomy using either an open or laparoscopic approach, and the pancreatic stump was closed with a stapling device [10]. Drain placement and number were determined at the surgeon′s discretion.

### 2.3. Postoperative Management

Two or three drains were placed near the pancreatic anastomosis, and the drain fluid amylase level was monitored until removal (typically by Postoperative Day [POD] 7). Octreotide was administered until POD 5, and oral intake commenced on POD 3 as tolerated. Clinically relevant postoperative pancreatic fistula (POPF) was defined according to the 2016 International Study Group on Pancreatic Fistula criteria [11]. Glycemic control consisted of sliding‐scale insulin during the early postoperative period, with adjustments directed by a diabetologist once oral intake resumed.

### 2.4. CGM

Glucose levels were continuously recorded from POD 1 for up to 14 days using the FreeStyle Libre Pro device (Abbott Diabetes Care Inc.). Data were analyzed using the FreeStyle Libre Pro software.

**Figure 1:**
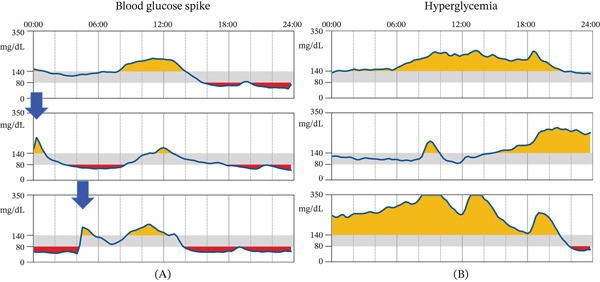
(A) Blood glucose spike: glucose > 7.8 mmol/L (140 mg/dL), followed by a decline to < 7.8 mmol/L within 2 h. (B) Postoperative hyperglycemia: glucose > 8.8 mmol/L (160 mg/dL) for > 6 h.

BGS was defined as a glucose level > 7.8 mmol/L (140 mg/dL), followed by a decline to < 7.8 mmol/L within 2 h (Figure 1A). Postoperative hyperglycemia was defined as a glucose level > 8.8 mmol/L (160 mg/dL) sustained for more than 6 h (Figure 1B). Nocturnal BGS/hyperglycemia was defined as respective events occurring between 23:00 and 07:00.

The target glucose range was 4.4–7.8 mmol/L (80–140 mg/dL). Time in range (TIR), time above range (TAR), and time below range (TBR) were calculated.

### 2.5. Statistical Analysis

The primary outcome was the association between BGS and postoperative complications, defined as Clavien–Dindo Grade ≥ II events. Continuous variables were compared using the Mann–Whitney *U* test and categorical variables using the chi‐squared test. All categorical variables are expressed as numbers (percentages), while continuous parameters are expressed as mean ± standard deviation. Variables with a *p* value < 0.10 in the univariate analysis were entered into a multivariate logistic regression model. A two‐sided *p* value < 0.05 was considered statistically significant. All statistical analyses were performed using EZR (Jichi Medical University, Tochigi, Japan). Due to the limited cohort size, the study was exploratory and lacked sufficient power to detect all potential outcomes.

### 2.6. Ethics Statement

Written informed consent was obtained from all participants prior to enrollment. The study was approved by the institutional review boards of both participating hospitals (Nakatsu Municipal Hospital: NMH2020041; Hiroshima Red Cross Hospital & Atomic Bomb Survivors Hospital: 2021‐035) and was conducted in accordance with the principles outlined in the Declaration of Helsinki.

## 3. Results

### 3.1. Patient Characteristics (PD vs. DP)

Of the 46 patients included, 34 underwent PD and 12 underwent DP. Baseline characteristics, including age, sex, body mass index, diabetes prevalence, history of upper abdominal surgery, and Charlson′s comorbidity index, were comparable between the PD and DP groups. Compared with those in the DP group, patients in the PD group had significantly lower preoperative albumin levels (38.0 vs. 42.0 g/L, *p* = 0.009), lower prognostic nutritional index (45.8 vs. 49.7, *p* = 0.046), higher incidence of postoperative complications, and longer length of hospital stay (Table 1).

**Table 1:** Baseline characteristics and postoperative glucose profiles of patients undergoing pancreatoduodenectomy or distal pancreatectomy.

Variables	Pancreatoduodenectomy (*n* = 34)	Distal pancreatectomy (*n* = 12)	*p* value
Preoperative factors			
Age (years)	69.5 ± 11.5	67.5 ± 10.7	0.600
Sex ratio: M/F	22/12	8/4	1.000
Body mass index (kg/m^2^)	22.7 ± 3.7	22.1 ± 2.7	0.649
Diabetes mellitus, *n*	6	4	0.416
History of upper abdominal surgery, *n*	6	1	0.657
Charlson′s comorbidity index	3.6 ± 1.8	3.8 ± 2.0	0.728
Albumin (g/dL)	3.8 ± 0.5	4.2 ± 0.3	0.009
Lymphocyte count (/*μ*L)	1506.3 ± 524.8	1491.8 ± 519.1	0.935
Prognostic nutritional index	45.8 ± 6.1	49.7 ± 4.1	0.046
HbA1c (%)	6.0 ± 1.1	6.5 ± 1.8	0.215
Malignant disease, *n*	29	9	0.412
Operative factors			
Surgical time (min)	490.6 ± 111.4	274.7 ± 82.6	< 0.001
Blood loss (mL)	898.0 ± 617.3	550.4 ± 505.1	0.087
Blood transfusion, *n*	9	2	0.701
Soft pancreas, *n*	23	10	0.461
Pancreatic fistula risk score	17.5 ± 8.3	15.6 ± 8.4	0.504
Postoperative factors			
Oral intake start (POD)	8.1 ± 4.8	3.8 ± 1.5	0.004
Enteral nutrition, *n*	15	0	0.004
Complication rate (%)	24	3	0.015
Hospital stay (days)	34.4 ± 16.9	19.9 ± 15.3	0.012
Postoperative glucose profile			
Mean glucose (mmol/L)	6.7 ± 1.5	6.9 ± 1.0	0.724
Maximum glucose (mmol/L)	12.1 ± 3.8	13.2 ± 2.9	0.391
Minimum glucose (mmol/L)	3.5 ± 1.0	3.2 ± 0.9	0.376
Duration within normal glucose range (%)	68.5 ± 23.6	66.5 ± 15.6	0.787
Duration above normal glucose range (%)	21.5 ± 23.4	26.2 ± 17.6	0.530
Duration below normal glucose range (%)	9.9 ± 11.1	7.3 ± 8.8	0.451
Postoperative hyperglycemia, *n*	18	7	1.000
Nocturnal hyperglycemia, *n*	12	0	0.020
Blood glucose spike, *n*	23	12	0.043
Nocturnal blood glucose spike, *n*	14	0	0.009
Insulin used (U)	52.6 ± 77.3	21.9 ± 27.5	0.188

*Note:* Data are presented as mean ± standard deviation unless otherwise indicated.

Abbreviations: HbA1c, hemoglobin A1c; M/F, male/female; POD, postoperative day.

### 3.2. Postoperative Glucose Profiles (PD vs. DP)

Mean, maximum, and minimum glucose levels, as well as TIR, TAR, and TBR, did not differ significantly between the groups (Table 1). However, nocturnal hyperglycemia unrelated to meals was more frequent after PD (12 of 34 patients, 35% vs. 0 of 12, 0%, *p* = 0.020). In contrast, postprandial BGS (including spikes > 11.1 mmol/L [200 mg/dL]) was more common after DP (6 of 12 vs. 5 of 34, *p* = 0.022). BGS occurred in all patients who underwent DP (Table 1).

### 3.3. Postoperative Complications After PD

Postoperative complications occurred in 24 of 34 patients who underwent PD (70.6%), of whom 17 had major complications (Clavien–Dindo Grade ≥ III) (Table 2). POPF occurred in 11 patients (32.3%) and pseudoaneurysms in three patients (8.8%). No deaths were recorded.

**Table 2:** Postoperative complications after pancreatoduodenectomy.

Complication	*n*	Clavien–Dindo classification
Septic shock	1	IVa
Arterial thrombus	1	IVa
Postoperative bleeding	1	IIIb
Wound dehiscence	1	IIIb
POPF	11	IIIa
Pseudoaneurysm	3	IIIa
Cholangitis	4	II
Ileus	2	II
Undernutrition	1	II

Abbreviation: POPF, postoperative pancreatic fistula.

### 3.4. Patient Characteristics (No‐Complication vs. Complication)

Patients who underwent PD were divided into no‐complication and complication subgroups. Patients who developed complications had a higher prevalence of soft pancreas (20 of 24, 83% vs. 3 of 10, 30%, *p* < 0.005), a higher pancreatic fistula risk score (19.9 vs. 11.9, *p* = 0.009), and a longer postoperative hospital stay (38.9 vs. 22.8 days, *p* = 0.010) than did those without complications (Table 3).

**Table 3:** Characteristics and postoperative glucose profiles of patients who underwent pancreatoduodenectomy with and without complications.

Variables	Without complications (*n* = 10)	With complications (*n* = 24)	*p* value
Preoperative factors			
Age (years)	69.2 ± 10.0	69.6 ± 12.2	0.271
Sex ratio: M/F	5/5	17/7	0.923
Body mass index (kg/m^2^)	23.6 ± 3.3	22.3 ± 3.9	0.374
Diabetes mellitus, *n*	3	3	0.328
History of upper abdominal surgery, *n*	2	4	1.000
Charlson′s comorbidity index	3.8 ± 2.0	3.5 ± 1.7	0.705
HbA1c (%)	6.5 ± 1.5	5.7 ± 0.8	0.103
Albumin (g/dL)	3.9 ± 0.4	3.8 ± 0.5	0.798
Lymphocyte count (/*μ*L)	1495.4 ± 302.1	1510.8 ± 599.5	0.939
Prognostic nutritional index	46.1 ± 3.6	45.7 ± 7.0	0.866
Malignant disease, *n*	8	19	1.000
Operative factors			
Surgical time (min)	535.4 ± 102.2	471.9 ± 111.8	0.132
Blood loss (mL)	879.6 ± 554.7	905.7 ± 652.8	0.913
Blood transfusion, *n*	2	7	0.692
Soft pancreas, *n*	3	20	< 0.005
Pancreatic fistula risk score	11.9 ± 7.2	19.9 ± 7.7	0.009
Postoperative factors			
Oral intake start (POD)	6.7 ± 1.9	8.7 ± 5.5	0.265
Total parenteral nutrition, *n*	1	8	0.225
Enteral nutrition, *n*	5	10	0.718
Fever, *n*	1	15	0.008
Hospital stay (days)	22.8 ± 5.9	38.9 ± 18.0	0.010
Postoperative glucose profiles			
Mean glucose (mmol/L)	7.0 ± 2.4	6.6 ± 0.9	0.453
Maximum glucose (mmol/L)	12.9 ± 5.5	12.0 ± 3.0	0.803
Minimum glucose (mmol/L)	3.5 ± 1.0	3.6 ± 1.0	0.839
Duration within normal glucose range (%)	65.8 ± 33.5	69.7 ± 18.9	0.671
Duration above normal glucose range (%)	23.9 ± 35.2	20.5 ± 17.2	0.708
Duration below normal glucose range (%)	10.3 ± 10.0	9.8 ± 11.7	0.908
Postoperative hyperglycemia, *n*	3	12	1.000
Nocturnal hyperglycemia, *n*	1	11	0.061
Blood glucose spike, *n*	2	21	< 0.001
Nocturnal blood glucose spike, *n*	0	7	0.078
Insulin used (U)	58.3 ± 71.7	54.2 ± 83.1	0.896

*Note:* Data are presented as mean ± standard deviation unless otherwise indicated.

Abbreviations: HbA1c, glycated hemoglobin; M/F, male/female; POD, postoperative day.

### 3.5. Postoperative Glucose Profiles (No‐Complication vs. Complication)

As shown in Table 3, mean, maximum, and minimum glucose levels, as well as TIR, TAR, and TBR, were similar between the two groups. However, BGS was significantly more frequent in patients with complications (21 of 24, 87.5% vs. 2 of 10, 20%, *p* < 0.001). Insulin use during CGM did not differ significantly between the two groups. Although not statistically significant, BGS tended to occur before the diagnosis of complications (6.8 ± 3.6 vs. 9.3 ± 4.8 POD, *p* = 0.077; data not shown).

### 3.6. Predictors of Morbidity

Multivariate analysis identified postoperative BGS as the only independent predictor of complications after PD (hazard ratio 30.2, 95% confidence interval 2.12–430; *p* = 0.012) (Table 4).

**Table 4:** Multivariate analysis of risk factors for postoperative complications.

Variables	Odds ratio (95% CI)	*p* value
Postoperative fever	17.5 (0.979–313)	0.052
Pancreatic fistula risk score > 15	1.92 (0.13–27.9)	0.631
Blood glucose spike	30.2 (2.12–430)	0.012

Abbreviation: CI, confidence interval.

## 4. Discussion

Postoperative complications are more frequent in patients with impaired glucose tolerance, whereas infectious complications are less common in patients with diabetes exhibiting good postoperative glycemic control [12–16]. Pancreatic resection, closely linked to glycemic regulation, is often associated with diabetes mellitus (DM). The development or worsening of DM after resection is very common, with risk varying by procedure: DP carries a higher risk of new‐onset postoperative diabetes, whereas PD has been reported to improve pre‐existing diabetes in some patients [17–20]. In this study, postoperative BGS, rather than absolute hyperglycemia, was strongly associated with morbidity after PD. These findings suggest that glycemic variability is a clinically relevant marker of postoperative stress and may contribute to the development of complications.

In the DP group, BGS was universal and often severe, likely reflecting postoperative endocrine insufficiency due to islet loss, particularly from the pancreatic tail, where islet density is the highest [21, 22]. In contrast, BGS was less common overall after PD but was significantly associated with severe complications, suggesting that inflammatory and metabolic stress may trigger glycemic excursions. Reports that diabetes improves after bariatric procedures, such as Roux‐en‐Y gastric bypass and Billroth II gastrojejunostomy, support this hypothesis [23–25], as the anatomical changes after PD resemble those observed in bariatric surgery and may alter hormone dynamics. In addition, the presence of a soft pancreas, a known risk factor for POPF and other complications, was more frequent in the complication group, which may contribute to glycemic variability through pancreatic enzyme leakage and subsequent inflammatory responses.

The association between BGS and complications after PD suggests a link between glycemic instability and systemic inflammation. Although not statistically significant, nocturnal BGS occurred more frequently in the complication group, implying dysregulation of basal insulin secretion, possibly exacerbated by surgical stress and impaired pancreatic function. The trend toward higher nocturnal hyperglycemia may reflect the cumulative effects of inflammation and stress‐induced hyperglycemia, both of which adversely influence healing and recovery [26–30].

Importantly, BGS often preceded the clinical recognition of complications, highlighting its potential value as an early warning biomarker. The significantly higher rate of BGS in the complication group underscores its pathological relevance beyond simple glucose level elevation. Although the underlying mechanism is unclear, acute fluctuations in glucose levels may increase endothelial stress, thereby increasing the risk of microvascular injury. This finding aligns with previous findings that have demonstrated an association between glycemic excursions and endothelial dysfunction and oxidative stress, both of which impair tissue repair and recovery [4]. Monitoring BGS in the early postoperative period may, therefore, represent a preventive strategy against severe complications.

Multivariate analysis confirmed BGS as the strongest independent predictor of postoperative morbidity, with a hazard ratio of 30.2. These findings support the integration of CGM into postoperative care after pancreatectomy. Early identification of BGS may prompt timely diagnostic work‐up and intervention, potentially improving outcomes. Furthermore, conventional insulin protocols may be insufficient to prevent BGS, underscoring the need for more proactive and individualized glycemic management strategies, including continuous insulin infusion or agents targeting postprandial glycemic control [31].

Several limitations should be acknowledged. First, the sample size was small with an imbalance between the two groups, and although the study was conducted at two centers, the overall cohort remained limited, which may restrict statistical power and generalizability. Second, patients were enrolled over a relatively long period, potentially introducing heterogeneity in perioperative management. Third, surgical approaches differed between groups (open PD vs. mixed open/laparoscopic DP), and robotic surgery was not included, which may limit generalizability. Finally, as this was an observational study, causality between BGS and postoperative complications cannot be definitively established. Larger, multicenter studies and mechanistic investigations are required to validate these results.

## 5. Conclusions

Postoperative BGS is an independent predictor of morbidity after PD and often precedes the clinical recognition of complications. Incorporating CGM into postoperative care and developing targeted strategies to reduce BGS may help lower complication rates and improve outcomes in patients undergoing pancreatic surgery.

## Author Contributions

All authors were involved in the study design. Shigeyuki Nagata completed the data analysis and drafted the original manuscript. Shigeyuki Nagata, Seigo Korematsu, Hiroyuki Orita, and Takashi Maeda interpreted the data.

## Funding

No external funding was granted for this study; it was independently funded by the authors.

## Disclosure

All the authors have read and approved this manuscript.

## Conflicts of Interest

The authors declare no conflicts of interest.

## Data Availability

The datasets of this study are available from the corresponding author upon reasonable request.
